# High-Power GaN-Based Vertical Light-Emitting Diodes on 4-Inch Silicon Substrate

**DOI:** 10.3390/nano9081178

**Published:** 2019-08-17

**Authors:** Qiang Zhao, Jiahao Miao, Shengjun Zhou, Chengqun Gui, Bin Tang, Mengling Liu, Hui Wan, Jinfeng Hu

**Affiliations:** 1 The Institute of Technological Sciences, Wuhan University, Wuhan 430072, China; 2 Center for Photonics and Semiconductors, School of Power and Mechanical Engineering, Wuhan University, Wuhan 430072, China; 3 State Key Laboratory of Applied Optics, Changchun Institute of Optics, Fine Mechanics and Physics, Chinese Academy of Sciences, Changchun 130033, China

**Keywords:** VLED, silicon substrate, current spreading, Au–In eutectic bonding, laser lift-off

## Abstract

We demonstrate high-power GaN-based vertical light-emitting diodes (LEDs) (VLEDs) on a 4-inch silicon substrate and flip-chip LEDs on a sapphire substrate. The GaN-based VLEDs were transferred onto the silicon substrate by using the Au–In eutectic bonding technique in combination with the laser lift-off (LLO) process. The silicon substrate with high thermal conductivity can provide a satisfactory path for heat dissipation of VLEDs. The nitrogen polar n-GaN surface was textured by KOH solution, which not only improved light extract efficiency (LEE) but also broke down Fabry–Pérot interference in VLEDs. As a result, a near Lambertian emission pattern was obtained in a VLED. To improve current spreading, the ring-shaped n-electrode was uniformly distributed over the entire VLED. Our combined numerical and experimental results revealed that the VLED exhibited superior heat dissipation and current spreading performance over a flip-chip LED (FCLED). As a result, under 350 mA injection current, the forward voltage of the VLED was 0.36 V lower than that of the FCLED, while the light output power (LOP) of the VLED was 3.7% higher than that of the FCLED. The LOP of the FCLED saturated at 1280 mA, but the light output saturation did not appear in the VLED.

## 1. Introduction

GaN-based light-emitting diodes (LEDs) have attracted widespread concern for automotive front lighting, solid-state lighting, urban landscape lighting, backlights for liquid crystal displays (LCDs), visible light communications, and so forth [1,2,3,4,5,6,7,8,9]. Currently, it is significantly crucial to drive LEDs at high current densities for the demand of high-power LED. However, at high current densities, external quantum efficiency (EQE) of LEDs exhibits a reduction partly owing to non-uniform carrier distribution [10,11]. In addition, both n-electrode and p-electrode are located on the same side of the flip-chip LED (FCLED) and top-emitting LED (TELED), resulting in severe current crowding around electrodes [12,13,14]. Moreover, the FCLED and TELED suffer from severe heat conducting problems due to the poor thermal conductivity of insulting sapphire substrate [15,16]. Consequently, high junction temperature induced by heat accumulation further impacts the optical and electrical properties of FCLEDs and TELEDs [17,18]. Furthermore, a portion of the active region has been etched in order to form an n-type contact, which decreases the active region area and consequently reduces the light output power (LOP) of FCLEDs and TELEDs [19]. The VLEDs technology is adopted as a practical approach to overcome these problems [20,21,22,23].

Recently, different substrates and bonding techniques have been used to improve the performance of VLED. For example, Wu et al. investigated the effect of electroplated copper substrate on the optical and electrical properties of VLEDs. The results showed that using electroplated copper as a heat dissipation substrate could release inherent joule heating, resulting in superior optical, electrical and heat dissipation performance [24,25,26]. Tran et al. demonstrated that metal-alloyed substrate could provide excellent heat dissipation performance [27,28,29]. However, the deformation of metal substrate increases the difficulty of laser scribing. Wong et al. transferred the GaN epitaxial layer onto GaAs and polymer substrates using a low-temperature Pd–In bonding technique [30]. However, GaAs and polymer substrates exhibit poor thermal dissipation properties. Liu et al. fabricated GaN-based blue VLEDs by using a Sn fusion bonding technique [31]. However, the stability of VLEDs with Sn as a bonding layer is poor at high temperatures [32].

In this study, GaN-based high-power VLEDs were fabricated on 4-inch silicon substrate by using Au–In eutectic bonding technique in combination with laser lift-off (LLO) process. The nitrogen polar n-GaN surface was textured by KOH solution, which not only improved the light extract efficiency (LEE) but also broke down the Fabry–Pérot interference, which resulted in a near Lambertian emission pattern. In addition, the ring-shaped n-electrode was formed to improve current spreading of VLED. Furthermore, numerical investigations on current spreading, heat dissipation and LEE of FCLEDs and VLED were performed using SimuLED, COMSOL multi-physics and finite-difference time-domain (FDTD) software. The optical and electrical properties of FCLEDs and VLEDs were experimentally compared. We found the current spreading and thermal dissipation abilities of VLEDs were superior to those of FCLEDs, which rationally explained the lower forward voltage and higher light output power (LOP) of VLEDs.

## 2. Experiments

The GaN-based LED structure was grown on a 4-inch sapphire substrate using metal-organic chemical vapor deposition (MOCVD) method. The LED epitaxial structure consists of a 25-nm-thick low temperature GaN nucleation layer, a 3.0-μm-thick undoped GaN layer, a 2.5-μm-thick n-GaN layer (silicon doping = 5 × 10^18^ cm^−3^), a 12-pair of In_0.16_Ga_0.84_N (3 nm)/GaN (12 nm) multiple quantum wells (MQWs), a 50-nm-thick p-Al_0.2_Ga_0.8_N electron blocking layer (Mg doping = 1.5 × 10^20^ cm^−3^), a 21-nm-thick p-AlGaN/GaN superlattices, and a 150-nm-thick p-GaN layer (Mg doping = 1.0 × 10^20^ cm^−3^). The GaN quantum barrier was grown at 870 °C, then the reactor temperature was decreased to 780 °C for the growth of the In_0.16_Ga_0.84_N quantum well. The LED wafer was annealed at 750 °C in N_2_ atmosphere to activate Mg in the p-GaN layer.

**Figure 1:**
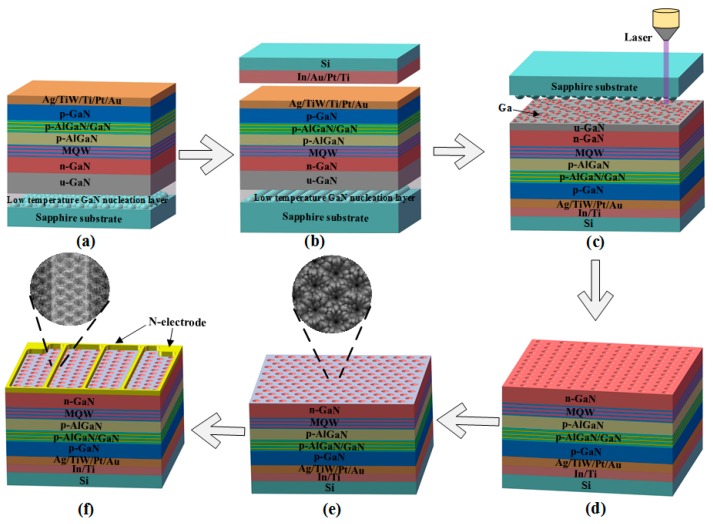
Schematic illustration of fabrication process for high-power vertical light-emitting diode (VLED): (**a**) Ag/TiW/Ti/Pt/Au was deposited onto p-GaN layer. (**b**) Light-emitting diode (LED) wafer was bonded to silicon substrate using Au–In eutectic bonding technique. (**c**) Patterned sapphire substrate (PSS) was removed using the laser lift-off (LLO) process. (**d**) Ga residual was removed by HCl solution and u-GaN was then etched by using inductively coupled plasma (ICP) etching process. (**e**) The n-GaN surface was roughened by KOH solution. (**f**) Cr/Pt/Au metal electrode was deposited onto the n-GaN layer.

The fabrication process of VLEDs consists of following steps: (a) Ag/TiW/Ti/Pt/Au (120 nm/80 nm/80 nm/80 nm/80 nm) was deposited onto p-GaN as p-type ohmic contact followed by thermal annealing in N_2_ ambient at 600 °C for 20 min (Figure 1a). (b) Ti/Pt/Au/In (80 nm/80 nm/80 nm/2 μm) was then deposited on Si-wafer. Next, the 4-inch LED wafer was bonded to the Si-wafer at 230 °C under 2000 kg pressure for 40 min. (c) The LLO process was employed to remove the patterned sapphire substrate (PSS) by using a 248-nm krypton fluoride (KrF) laser with power density of 0.9 J/cm^2^. (d) The HCl:H_2_O (1:1) solution was used to etch the residual Ga on u-GaN. After removing the residual Ga, an inductively coupled plasma (ICP) etching process was employed to etch the u-GaN until the n-GaN layer was exposed. (e) The n-GaN surface was roughened by a KOH wet chemical etching at 70 °C. (f) Cr (20 nm)/Pt (50 nm)/Au (1.5 μm) multilayers were deposited on the n-GaN layer as an n-electrode. For comparison, the FCLED with the same epitaxial structure was also fabricated. The detailed fabrication process of FCLEDs was shown in our previous paper [33]. The size of the VLED was 1000 × 1000 μm^2^, and the peak emission wavelength was 453 nm. The LOP versus current (L–I) and current versus voltage (I–V) characteristics of VLEDs and FCLEDs were measured by using an integrating sphere and semiconductor parameter analyzer Keysight B2901A (TI, Dallas, TX, USA). All relevant chemical reagents, including potassium hydroxide (KOH), hydrochloric acid (HCl), were obtained from Shenshi Chemical Corporation (Wuhan, China).

## 3. Results and Discussion

**Figure 2:**
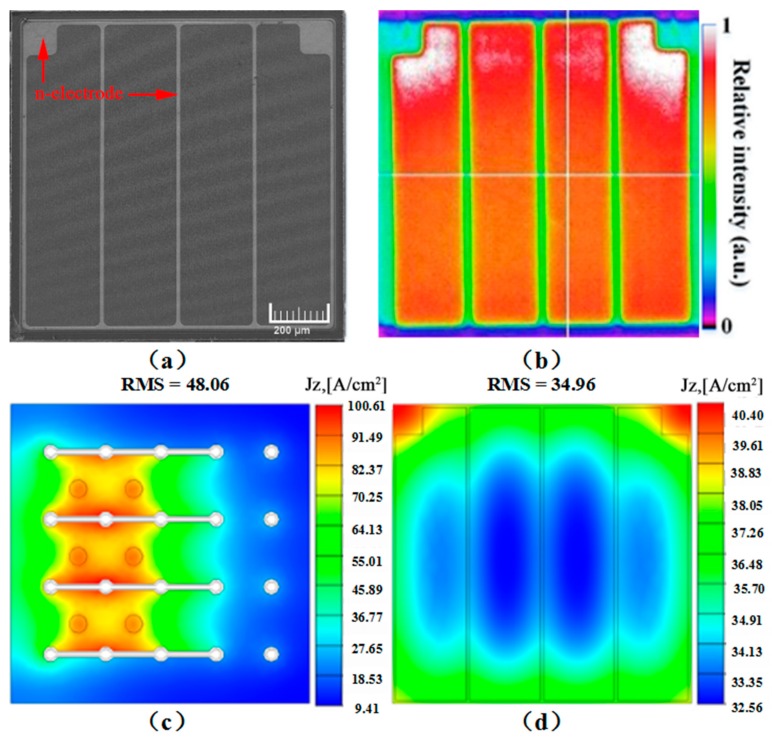
(**a**) Top-view SEM image of VLED showing the n-electrode. (**b**) Measured light emission intensity distributions of VLED at 350 mA. (**c**) Current density distribution in active region of VLED at 350 mA under 27 °C ambient temperature. (**d**) Current density distribution in active region of FCLED at 350 mA under 27 °C ambient temperature.

The top-view SEM image of the fabricated VLED was shown in Figure 2a. As shown in Figure 2a, the ring-shaped n-electrode was uniformly distributed over the entire VLED. Additionally, the n-GaN surface was roughened to improve the LEE of the VLED. We measured the light emission image of the VLED using a calibrated charge-coupled device (CCD) camera at 350 mA. Figure 2b shows the light emission intensity distribution of the VLED driven at an injection current of 350 mA. It was found that the light emission intensity over the entire active region of the VLED was strong except for the area covered by n-electrode, as shown in Figure 2b. Figure 2c–d show the current density distribution of the FCLED and VLED at 350 mA. The root mean square (RMS) and maximum current density of the FCLED in an active region are 48.06 A/cm^2^ and 100.61 A/cm^2^, respectively. Additionally, it can be observed that the current is crowded around the n-contact fingers of FCLED, resulting in an inhomogeneous current distribution. Compared to the FCLED, the RMS of the VLED in the active region is reduced to 34.96 A/cm^2^. The maximum current density of the VLED is only 49.29 A/cm^2^, which is much less than that of the FCLED. Therefore, it could be concluded that the current spreading of VLEDs was superior to that of FCLEDs. Table 1 shows the current density distributions of FCLEDs and VLEDs at 200, 350, 500, and 700 mA.

**Figure 3:**
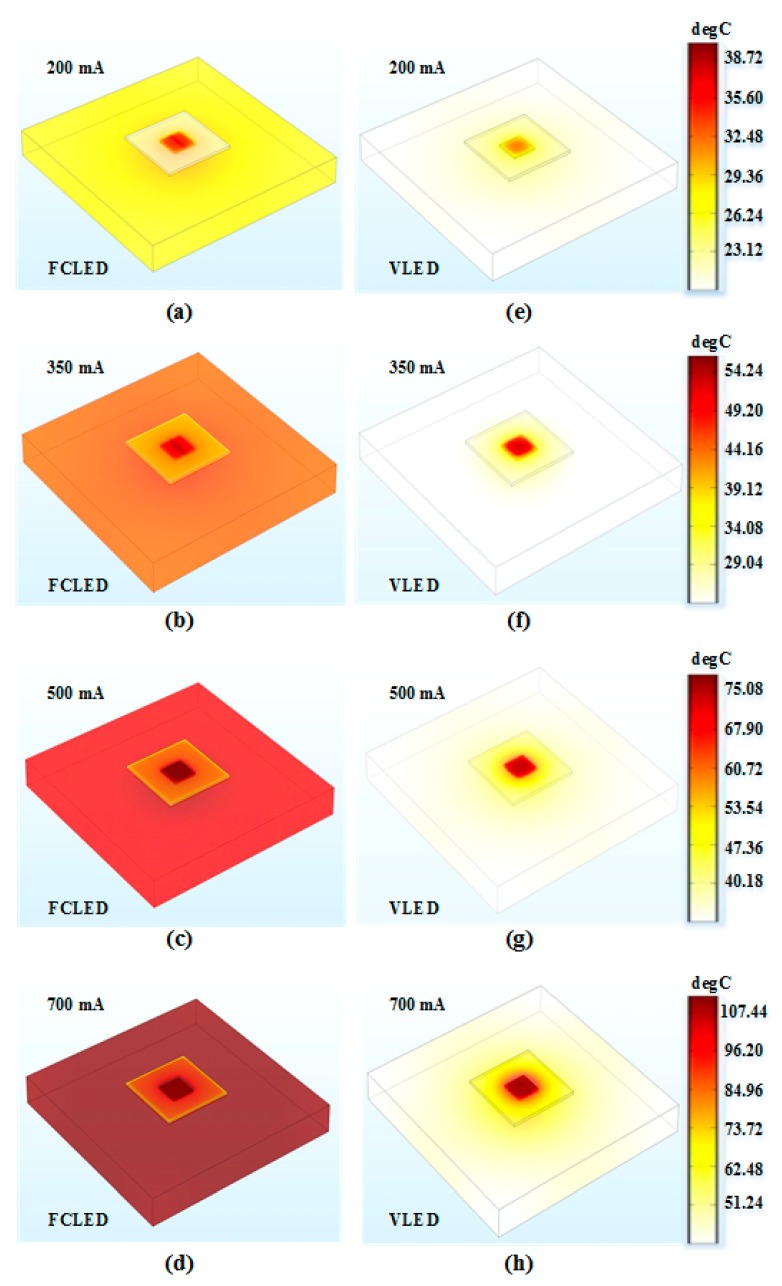
(**a**–**d**) Temperature distributions of FCLEDs. At 200, 350, 500, and 700 mA, the maximum temperatures of FCLEDs were 35.90, 53.71, 74.52, and 107.41 °C, respectively. (**e**–**h**) Temperature distributions of VLEDs. At 200, 350, 500 and 700 mA, the maximum temperatures of VLED were 31.78, 44.83, 59.90, and 83.11 °C, respectively.

Thermal analysis of the FCLED and VLED was performed using COMSOL Multiphysics. The FCLEDs and VLEDs are fixed onto an Al heat sink and then encapsulated with epoxy resin. The specific heat, thermal conduction coefficient, and density of the sapphire substrate are 730 J/kg·K, 35 W/m·K, and 3.96 g/cm^3^ [34] respectively, while the corresponding values of the silicon substrate are 700 J/kg·K, 149 W/m·K, and 2.33 g/cm^3^, respectively. According to the material parameters, numerical investigation on the temperature distribution of FCLEDs and VLEDs was performed at room temperature (27 °C). The simulation results are illustrated in Figure 3a–h. At 200, 350, 500, and 700 mA, the maximum temperatures of FCLEDs were 35.90, 53.71, 74.52, and 107.41 °C, respectively. Due to the low resistance of the vertical current path, the VLED generated less heat. Furthermore, the VLED exhibits superior heat dissipation due to the high thermal conductivity of silicon substrate. Hence, the corresponding maximum temperatures of the VLED were reduced to 31.78, 44.83, 59.90, and 83.11 °C, respectively. Table 2 shows the maximum and minimum temperatures of FCLEDs and VLEDs at 200, 350, 500, and 700 mA.

**Figure 4:**
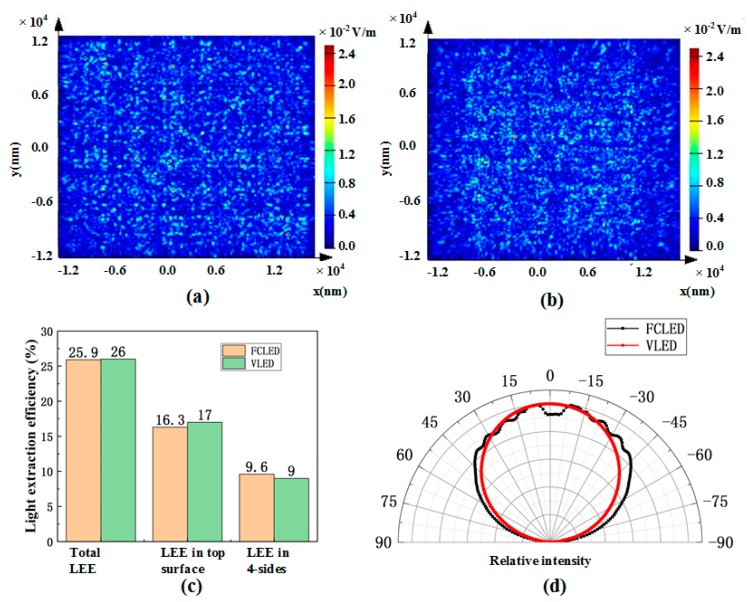
(**a**) Normalized electric field distribution from top surface of the FCLED. (**b**) Normalized electric field distribution from top surface of the VLED. (**c**) Light extract efficiency (LEE) of each face (top and four-sides) of FCLED and VLED. (**d**) Far-filed radiation pattern of FCLED and VLED at 350 mA.

The LEE of FCLEDs and VLEDs was calculated using the finite-difference time-domain (FDTD). The simulation model was built based on the above-described device structure and scaled to the size of 20 μm × 20 μm (L × W) considering the computational capacity. Transverse electric (TE) polarized point sources were positioned in the center region of the MQWs and the emission wavelength was set to 453 nm. Figure 4a,b shows the normalized electric field intensity distribution of FCLEDs and VLEDs. The electric field distribution of FCLEDs was broader than that of VLEDs, which indicated a more divergent emission from the top-emitting surface of the FCLED. Figure 4c shows the calculated LEE of different emitting surfaces. The LEE in 4-sides of a VLED (9.0%) was lower than that of a FCLED (9.6%). However, the LEE in the top surface of a VLED with textured n-GaN surface (17.0%) was higher than that of a FCLED (16.3%). The total LEE of FCLEDs (25.9%) and VLEDs (26.0%) was almost the same. In Figure 4d, the far-field radiation pattern of the FCLED is featured with Fabry–Pérot fringes due to optical cavity effects. In contrast, the VLED shows a near Lambertian emission pattern, revealing that the Fabry–Pérot interference was broken down in the VLED. The absence of Fabry–Pérot interference in the VLED was due to the textured n-GaN surface. Additionally, the emission of the VLED was more concentrated in the top surface than that of the FCLED as indicated by the stronger axial emission intensity in the VLED.

**Figure 5:**
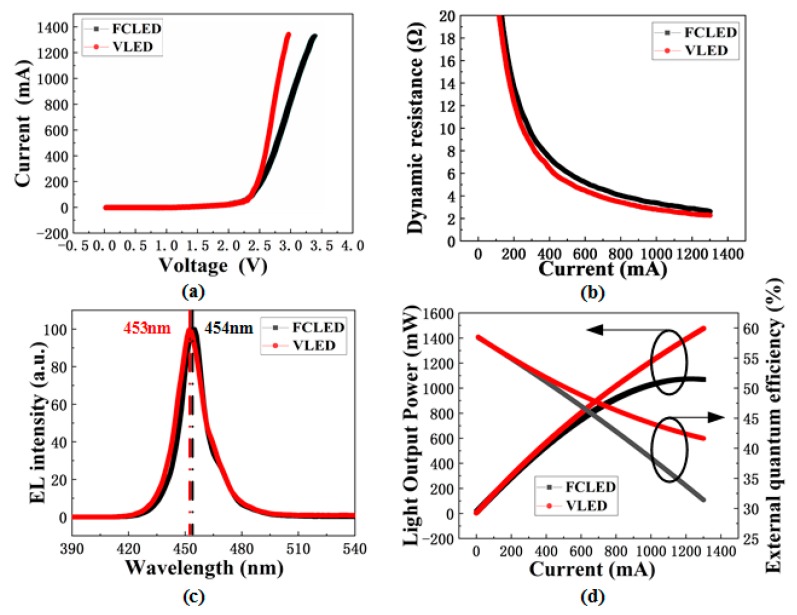
(**a**) Forward voltage versus current for high-power VLEDs and FCLEDs. (**b**) Dynamic resistance versus current for high-power VLEDs and FCLEDs. (**c**) Electroluminescence spectra of high-power VLEDs and FCLEDs measured at 350 mA. (**d**) Light output power (LOP) and external quantum efficiency (EQE) versus current for high-power VLEDs and FCLEDs.

Figure 5a shows the forward voltage versus current for a high-power VLED and FCLED. At an injection current of 350 mA, the forward voltage of VLED was 2.60 V, which was 0.29 V lower than that of the FCLED (2.89 V). Figure 5b shows the dependence of dynamic resistance on the injection current for the VLED and FCLED. The series resistance of VLED deduced from the dynamic resistance curve was 2.25 Ω, which was 0.47 Ω lower than that of the FCLED (2.72 Ω). The lower series resistance of VLED was attributed to the vertical current path and the less current crowding effect in proximity of the metal electrodes. The electroluminescence spectra of high-power VLEDs and FCLEDs measured at 350mA was shown in Figure 5c. The peak emission wavelength of the VLED and FCLED were 453 nm and 454 nm, respectively. The small difference in electroluminescence spectra may arise from reduced quantum confined Stark effect (QCSE) in VLEDs since the compressive strain in GaN epilayers was mitigated with the removal of the sapphire substrate by LLO. Figure 5d shows the LOP versus current for the VLED and FCLED. At 350 mA, the LOPs of FCLEDs and VLEDs were respectively 479.9 and 497.6 mW. The EQE of blue LEDs is described by

EQE = PhvIe = PλI × ehc = Pλ1240I

where *P* is the light output power (LOP) of LEDs, *λ* is the light emission wavelength, *I* is the injection current, *e* is the elementary charge, *h* is the Planck constant, *c* is the speed of light, and *v* is the frequency of light. The light emission wavelength of the blue LEDs was 453 nm. The corresponding EQEs of FCLEDs and VLEDs were 51.6% and 52.1%, respectively. The LOP of FCLEDs saturated at 1280 mA, but the light output saturation did not appear in VLEDs owing to the better heat dissipation ability and reduced current crowding around electrodes.

## 4. Conclusions

In summary, we compared the optical, electrical, and thermal properties of VLED and FCLED. The high-power VLED on silicon substrate was fabricated by using LLO and Au–In eutectic bonding technique. It was found that the current spreading and thermal dissipation of VLEDs were superior to these of FCLEDs. At 350 mA, the maximum current density and maximum temperature of a FCLED were 100.61 A/cm^2^ and 53.71 °C, and the corresponding maximum values of a VLED were reduced to be 49.29 A/cm^2^ and 44.83 °C. At 1280 mA, the LOP of the VLED (1457.4 mW) was 23.4% higher than that of the FCLED (1181.1 mW). The higher LOP of the VLED was attributed to the superior current spreading and better heat dissipation.

## Figures and Tables

**Table 1:** Current density distribution in the active region of high-power FCLEDs and VLEDs at 200, 350, 500, and 700 mA.

Current	200 mA		350 mA		500 mA		700 mA	
LED Type	FCLED	VLED	FCLED	VLED	FCLED	VLED	FCLED	VLED
Maximum current density (A/cm^2^)	56.41	22.76	100.61	49.29	146.13	58.15	211.05	82.13
Minimum current density (A/cm^2^)	6.18	18.84	9.41	32.66	12.5	46.40	16.38	64.62
Root mean square (RMS) value (A/cm^2^)	27.37	19.99	48.06	34.96	68.95	34.93	97.93	49.92

**Table 2:** Temperature distribution in the active region of FCLEDs and VLED at 200, 300, 500, and 700 mA, respectively.

Current	200 mA		350 mA		500 mA		700 mA	
LED Type	FCLED	VLED	FCLED	VLED	FCLED	VLED	FCLED	VLED
Maximum temperature (°C)	35.90	31.78	53.71	44.83	74.52	59.90	107.41	83.11
Minimum temperature (°C)	35.76	31.75	53.41	44.77	74.05	59.79	106.64	82.94
